# Safety-netting strategies for primary and emergency care: a codesign study with patients, carers and clinicians in Sweden

**DOI:** 10.1136/bmjopen-2024-089224

**Published:** 2024-08-05

**Authors:** Carolina Wannheden, Johanna Hagman, Sara Riggare, Karin Pukk Härenstam, Rita Fernholm

**Affiliations:** 1 Department of Learning, Informatics, Management and Ethics, Medical Management Centre, Karolinska Institutet, Stockholm, Sweden; 2 Stockholm Health Care Services, Academic Primary Healthcare Centre, Stockholm, Sweden; 3 Department of Women’s and Children’s Health, Participatory eHealth and Health Data Research Group, Uppsala University, Uppsala, Sweden; 4 Centre for Disability Studies, Uppsala University, Uppsala, Sweden; 5 Department of Pediatric Emergency, Astrid Lindgren Children's Hospital, Stockholm, Sweden; 6 Department of Neurobiology, Care Sciences and Society, Family Medicine and Primary Care, Karolinska Institutet, Stockholm, Sweden

**Keywords:** person-centered care, community-based participatory research, decision making, primary care, safety, emergency service, hospital

## Abstract

**Abstract:**

**Objectives:**

To codesign safety-netting strategies for primary and emergency care settings by integrating the experiences and ideas of patients, carers and clinicians.

**Design:**

A codesign process involving two focus group discussions, eight individual interviews and five workshops. All sessions were audio recorded and transcribed verbatim. Data were analysed using qualitative content analysis and reported using the Consolidated criteria for Reporting Qualitative research guidelines.

**Setting:**

Primary and emergency care in Sweden, focusing on the Stockholm region.

**Participants:**

7 (5 women) individuals with patient expertise, 1 (man) individual with carer expertise, 18 (12 women) individuals with clinical expertise.

**Results:**

Three main categories reflecting strategies for applying safety-netting were developed: first, *conveying safety-netting advice*, which involves understanding patient concerns, tailoring communication and using appropriate modalities for communicating; second, *ensuring common understanding,* which involves summarising information, asking a teach-back question and anticipating questions post consultation; and third, *supporting safety-netting behaviour,* which involves facilitating reconsultation, helping patients and carers to navigate the health system and explaining the care context and its purpose.

**Conclusions:**

Our study highlights the collaborative nature of safety-netting, engaging both the clinician and patient, sometimes supported by carers, in an iterative process. Adding to previous research, our study also emphasises the importance of anticipating postconsultation inquiries and facilitating reconsultation.

**STRENGTHS AND LIMITATIONS OF THIS STUDY**

- The study combined expertise and experiences of patients, carers and clinicians through a comprehensive codesign process.
- Various data collection methods (focus group discussions, interviews and workshops) were triangulated.
- The study covered both primary and emergency care settings, although these were limited to the administrative unit of Region Stockholm in Sweden.

## Introduction

 Diagnostic errors are common causes of preventable harm in both primary and emergency care.^1^,^3^ Based on data from the USA, it has been estimated that at least 5% of adult patients are affected by diagnostic errors in outpatient care.^4^ Primary and emergency care settings are stressful environments in which clinicians must often make diagnostic decisions under time pressure. Poor communication between clinicians^5^ and between clinicians and patients^6^ has been identified as common contributing factors to preventable harm.

A promising approach to managing diagnostic errors and possibly preventing incorrect and delayed diagnoses is the communication of safety-netting advice to patients and family carers. Safety-netting has been defined as ‘[i]nformation shared with a patient or their carer, designed to help them identify the need to seek further medical help if their condition fails to improve, changes, or if they have concerns about their health’.^7^ In the UK, the provision of safety-netting advice in everyday clinical practice is recommended in national best practice guidelines.^8^ However, safety-netting advice is inconsistently used, in part due to the lack of appropriate guidance or time,^9 10^ and there is lacking consensus about what constitutes effective safety-netting.^11^ Nevertheless, some key features have been identified. For example, it has been suggested that the establishment of a mutual understanding between the clinician and patient is vital to safety-netting.^12 13^ Further, the provision of clear guidance has been highlighted as important to support patients in taking on increased responsibility.^11^ A realist review of how to effectively communicate safety-netting advice in primary care proposed a set of 15 recommendations for clinicians.^14^ Yet, integrating various recommendations in already time-pressured consultations may pose considerable challenge and there is still limited knowledge about successful communication strategies, especially as perceived by patients. A study performed in the context of lung cancer symptoms found that patients’ preferences may differ from clinicians’ expectations.^15^ While clinicians expressed worry of causing unnecessary concern by providing information about red flag symptoms, patients preferred active and clear safety-netting advice. This highlights the importance of including the patient voice in safety-netting studies, which aligns with an increased acknowledgement of the experiences and expertise of patients and public contributors in health service design^16 17^ and research.^18^ The aim of this study was to codesign safety-netting strategies for primary and emergency care settings by integrating the experiences and ideas of patients, carers and clinicians.

## Methods

### Swedish healthcare context

In Sweden, healthcare services are publicly financed, providing universal healthcare coverage. Responsibility for the services is delegated to the 21 regions and 290 municipalities, resulting in geographical differences.^19^ Primary care is usually the first point of contact during office hours, and patients can freely choose among providers. However, compared with most other European countries, a low proportion of citizens have a regular primary care doctor.^20^ In evenings and during weekends, patients can access out-of-hours offices staffed by primary care practitioners. In both settings, an average appointment lasts 10–15 min. The task of emergency departments is not specifically regulated and can to some extent be adapted by the regions.^19^ If patients seek emergency care for conditions that are not considered life-threatening, the waiting times are usually many hours and the amount of time spent with the clinician can be shorter than in primary and out-of-hours care. The provision of safety-netting advice is not specifically recommended in Swedish clinical practice guidelines. However, consultation techniques for person-centred care are increasingly used in routine practice.^21^

### Study design, setting and participants

We used an experience-based codesign approach^17^ to design safety-netting strategies for Swedish primary and emergency care settings, focusing on Region Stockholm. Participants were purposefully selected to achieve variation in terms of expertise and experience. All were approached by email. Patients and carers were recruited through different patient networks, aiming for knowledgeable and resourceful participants with varied healthcare experiences. Clinicians were recruited through the Swedish Association of General Practice and the Swedish Society for Pediatric Emergency Medicine, with the goal of achieving variation in practice setting (primary, emergency, out-of-hours care services), experience (residents and specialists) and specialties (adult and paediatric care). We recruited 26 participants: 7 (5 women) with patient expertise, 1 (man) with carer expertise and 18 (12 women) with clinical expertise. Among the clinicians, 13 were specialists (9 in general medicine, 3 in emergency medicine and 1 in internal medicine) and 5 were residents (4 in general medicine and 1 in emergency medicine). The age range was 46–58 (median 51) years among patients/carers and 21–61 (median 43) years among clinicians (see online supplemental appendix 1 for full participant details). Clinicians participated during work hours; patients/carers were reimbursed to compensate for loss of income, according to national guidelines.

### Codesign process

**Figure 1:**
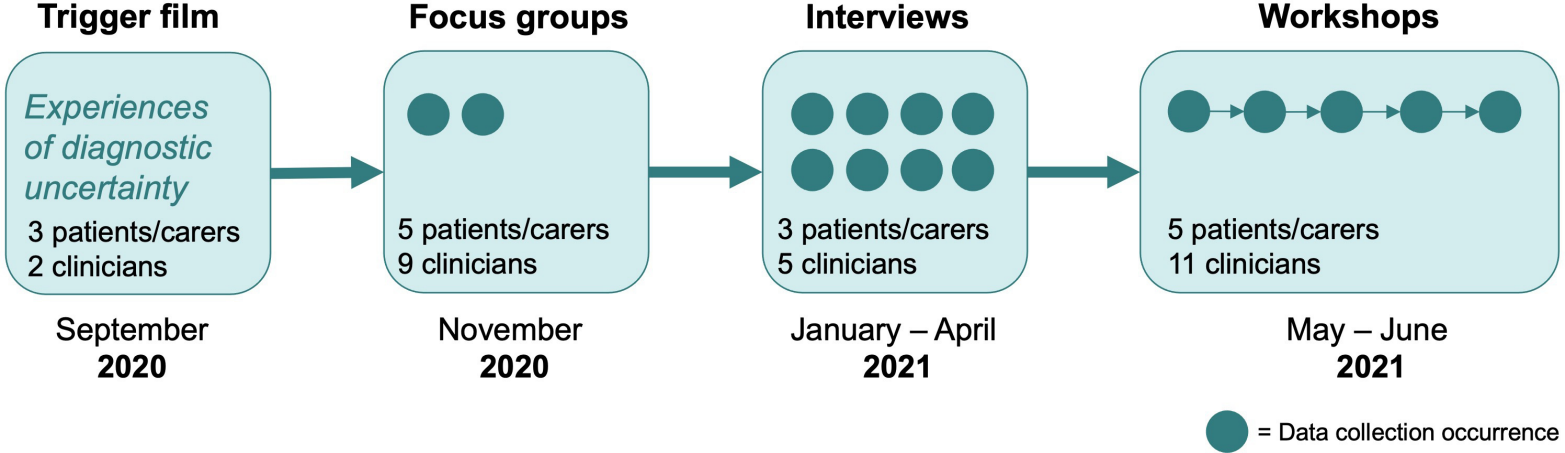
Codesign process involving in total 8 patients/carers and 18 clinicians.

The codesign process is illustrated in figure 1. Initially, we produced a short trigger film (9 min), which introduced the safety-netting concept and showed short filmed interviews with patients/carers (n=3) and clinicians (n=2) sharing their experiences of diagnostic uncertainty. The film was used to trigger discussions about safety-netting in two heterogeneous focus groups (ie, involving patients/carers and clinicians), followed by eight individual interviews. Results from the focus group discussions and interviews have already been published, describing preconditions for successful safety-netting.^12^ Codesign activities were performed in a series of five 1.5-hour workshops which were conducted online via Zoom. The overall aims of the workshop series, which were communicated to the participants, were to develop a common understanding of what safety-netting implies in the patient–clinician consultation, to set goals for what should be achieved with safety-netting, to develop strategies for integrating safety-netting in the consultation and to develop educational content for teaching safety-netting strategies to clinicians. The workshops were facilitated by RF, KPH, CW and one research assistant. Each workshop consisted of two moderated sessions (lasting 20–30 min each) in which the participants, separated into two heterogeneous groups, were tasked to discuss a specific question or to perform a design exercise. When the groups reconvened, facilitators summarised the main points from each group, followed by a brief discussion in the larger group. A detailed description of the aims, structure and content of each workshop session is provided in online supplemental appendix 2.

### Data collection and analysis

For details regarding the data collection and analysis of the focus group discussions and interviews, we refer to our previous publication.^12^ Of the workshop participants, all 5 of the patients/carers and 3 of the 11 clinicians had participated in a focus group discussion and/or interview; the remaining 8 clinicians had not participated in previous phases of the research project. All workshop sessions were audio recorded and video recorded and transcribed verbatim. We analysed the data inductively, using qualitative content analysis.^22^,^24^ One senior researcher (CW) and one research assistant (JH) coded and categorised the data. First, we individually read the transcripts to become familiar with the data and selected meaning units for coding. Next, we compared our selections and collaboratively created condensed meaning units and labelled them with codes in a Microsoft Excel spreadsheet. Thereafter, we transferred the data (ie, meaning units, condensed meaning units, codes and source identification) to a mind-mapping software (FreeMind version V.1.0.1^25^) for categorisation into a hierarchical structure. JH organised the data into categories and subcategories and refined these in iterations together with CW. After having created categories, we revisited our previous analysis of the focus group discussions and interviews. We found data that matched our created categories and merged these with our analysis; no additional categories needed to be created, suggesting that the workshops built on the ideas that had been raised in the focus group discussions and interviews. We discussed the final categorisation among all coauthors and refined the category labels together. In the final phase, illustrative quotes were selected and translated from Swedish to English, with minor adaptations to enhance their readability. The Consolidated criteria for Reporting Qualitative research guidelines were used for reporting.

### Patient and public involvement

This study involved a patient researcher (SR) who participated in the study design, the participant recruitment process, the final discussion of findings and the manuscript review. Other patients and carers participated in the codesign process.

## Results

**Figure 2:**
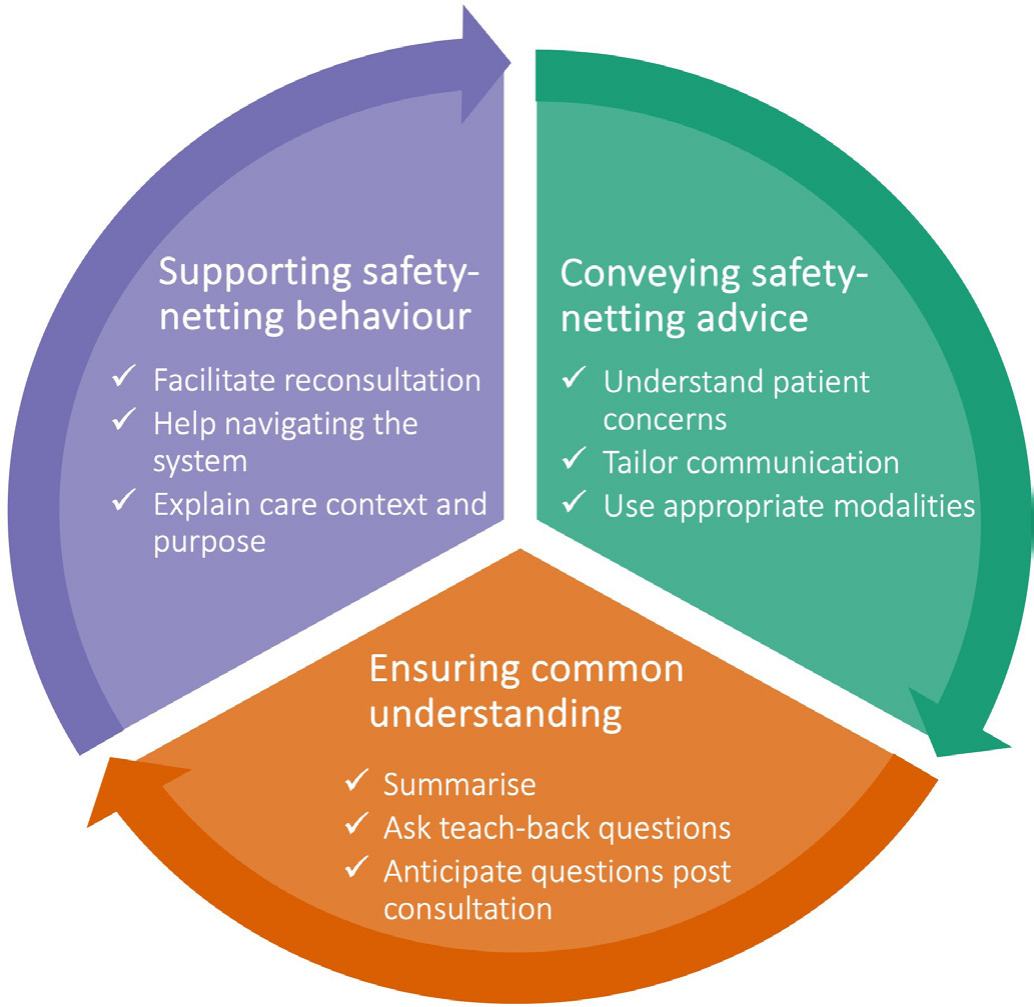
Categories (in bold) and subcategories (checkmarks) of strategies for applying safety-netting.

We created three main categories, each composed of three subcategories, reflecting strategies for applying safety-netting (figure 2).

### Conveying safety-netting advice

This category concerns strategies that clinicians should apply when conveying safety-netting advice to their patients.

#### Understand patient concerns

Participants explained that successful safety-netting is dependent on two-way interactions and collaboration between clinicians and patients. Discussions evolved specifically around strategies for enabling clinicians to increase their awareness of patients’ concerns and respond to these, as well as making patients feel seen and heard. Understanding and responding to patients’ concerns was considered a prerequisite for safety-netting. Someone stressed that “you also lay the foundations for it at the beginning of the meeting, by also asking ‘what are your expectations for this meeting?’” (workshop session 3, group B). One of the participants commented:

And I was thinking about that beautiful poem that I think many of us have seen in the staff room, Sören Kierkegaard’s “Till Eftertanke” [For Reflection]: that to be able to bring a person towards a goal, we first need to understand the person where she is. And that is a super important foundation for safety-netting, that we find a common platform for how this information should be delivered. (workshop session 3, group B)

#### Tailor communication

Participants agreed that there is an inherent power imbalance between patients and clinicians. As one of the participants put it, the basis for all patient–clinician conversations should be “that everyone is aware of the enormous hierarchical disadvantage that the patient finds themselves in, for obvious reasons” (workshop session 3). It is the responsibility of the clinician, therefore, to tailor communication so that it promotes a sense of openness. Clinicians should strive to be honest and transparent. They should reason at a level that is meaningful to the patient, an approach which requires adaptability and imagination. For example, clinicians described how they sometimes share their diagnostic reasoning process with a patient, which includes a disclosure of their uncertainty. In doing so, they said they aimed to use common terminology and calibrate the information load to the situation. The participants believed that patient information often needs to be adjusted to different target groups (eg, children, older adults, second language speakers), as well as to individual needs. From the perspective of patients and carers, someone emphasised that they wished the content of the discussion to be less clinical (eg, focusing on health parameters and medication) and more focused on the patient’s life situation ahead (eg, ‘how will this be for me?’ and ‘how can I live with this?’). As one of the clinicians remarked:

And that’s what’s difficult, I think, because the step to go from, that now I've finished my medical investigation, to adapting that information to the patient, it is, I don’t really have much training in that. (focus group 1)

#### Use appropriate modalities

Patients as well as clinicians expressed awareness of the fact that it is easy to forget or misunderstand advice. To avoid misunderstanding and enhance retention of information, participants suggested that it would be valuable to use a combination of modalities (eg, written and verbal information) when possible. While patients were of the opinion that written information that they could bring home and refer to is always of value, clinicians recognised that they adapt their mode of information delivery to an extent to the individual patient, but also on how much time they have. For example, some were more inclined to provide written information to patients with cognitive impairments, such as dementia or burnout, or if there was a language barrier. However, they also problematised that patients or their carers may suffer from unknown or hidden disabilities. Providing patients with standardised patient information (printed or online), or preprinted templates that could easily be complemented with patient-specific information, were suggested to save time. Other suggested modes for providing written advice were to encourage patients to take notes, as well as documenting safety-netting advice in the consultation notes that patients in Sweden have access to online (although with varying delay). Safety-netting in digital consultations (an online chat function) was also discussed, with some suggesting that communicating safety-netting advice was even easier in these scenarios, although there were other drawbacks (eg, limited ability to interpret tone and body language).

Facilitator: Do you think the same concept for safety-netting applies to digital consultations?

Participant: Yes, I think so. Yes, now I’m really sticking my neck out here, but I would argue that it’s actually somewhat easier. Because the patient gets this [information] in peace and quiet, in writing, has time to read, time to think it over, you can ask questions, if you’ve got any more questions… and all the patient has to do is to wait ten minutes to get an answer to their question. So there are significantly greater chances in the chat to pick up all that is difficult to miss in a time-limited physical consultation. (workshop session 8, group A)

### Ensuring common understanding

This category comprises strategies that can be adopted to ensure mutual understanding and the resolution of open questions.

#### Summarise

Participants emphasised the importance of summarising the discussion before ending the consultation. From a patient perspective, having a clinician summarise their concerns back to them helps them to know that the clinician captured everything as intended. Responding to this summary also enables the patient to comment or add information if necessary. One of the clinicians pointed out that, from their perspective, summarising a discussion enables them to evaluate whether the patient has followed their reasoning and if they have a shared understanding of the situation. A patient participant also noted that:

it helps me a lot if I at the end am asked if I would like to add anything…. So, then the doctor summarises… how the assessment has been reached and I can respond… yes, a few people do it. It’s very much appreciated. (workshop session 3, group A)

#### Ask teach-back questions

Another consultation tool that participants discussed was the teach-back method, implying that the patient repeats the safety-netting advice in their own words. It was described as a very effective method to ensure that the patient has correctly understood the safety-netting advice and to help them remember the content. Participants emphasised that this would be an important skill to teach clinicians in safety-netting education. Specifically, practical tips for how to introduce teach-back into a consultation without it coming across as a cross-examination were desired. One clinician said:

I’m a big advocate of getting patients to recap as well. With a 'What is your take-home message from today?' question. You have to find your own way of knowing what’s the right way to ask it, but it provides so much insight into what the patient is taking away with them of the plan you've set up. (workshop session 4, group A)

#### Anticipate questions post consultation

Accounting for the fact that questions may arise after the consultation has concluded was stressed as an important strategy to support safety-netting. One of the clinicians felt they needed to regularly remind themselves that actual care, and thereby also safety-netting, happens mainly after the consultation is over. To support patients in this phase, clinicians can encourage to ask questions. They can also proactively help patients by sharing common questions that may arise after their consultation. The participants also advocated clearly documented safety-netting advice which focused specifically on the plan going forward, which can be helpful for the patient as well as other healthcare professionals. While written documentation may answer some questions that arise, participants also discussed ways of responding to unanticipated questions that emerged after the consultation (eg, via chat or other text message services). One patient said:

Then I think that the doctor needs to be aware that I come up with a lot of questions when I’m in the car on my way home. And that must put high demands on you, but I think I have the right [to get answers]. Then it’s up to you to encourage me to ask questions: ‘Is there something you’re wondering about?’ And that you yourselves think, ‘But have you thought about…?’. So you could help us out there, so that the questions come up when we’ve got you in front of us, and not when we’re sitting in the car thinking ‘I should have asked this too’. (workshop session 3, group B)

### Supporting safety-netting behaviour

This category concerns strategies for adapting safety-netting advice to how healthcare is organised and supporting patients in implementing safety-netting behaviours.

#### Facilitate reconsultation

Making it easy for patients to reconsult or ask questions after a consultation was identified as an important facilitating condition for safety-netting behaviours. The participants discussed problems of ensuring relational continuity in primary care and emphasised that it is important that patients nevertheless feel welcome to return and have easy access to appropriate follow-up healthcare; patients should not feel that they need to force themselves into the healthcare system. Participants commented that this might be of particular importance if the safety-netting advice concerns symptoms that are diffuse or if the patient suffers from psychosocial problems that put them at risk of discrimination. One patient described getting additional advice as:

[a] feeling of needing to push yourself into healthcare. Half of my face felt like it was hanging off. [My chronic condition] and I have been living together for 10 years, so I know how it is. I knew it wasn't that. I got the advice to go home and rest and call in sick. ‘Rest, it’ll be fine.’ I sought emergency care again. It got worse. I experienced pain. (workshop session 1, group A)

A clinician stated that:

Patients should always feel welcome back; the question is who is the most suitable person to welcome them back? (workshop session 1, group B)

#### Help navigating the system

The participants discussed the challenge for patients to know at which level they should seek care. By way of illustrating how complex the care system was, one clinician exclaimed that “it’s insanely difficult, even I as a clinician can’t find my way around” (workshop session 1, group B). To make it easier for patients, participants agreed that clinicians should specify a plan, indicating at which level patients should seek care again if needed. Everyone agreed that it could be difficult to create a general rule for when to seek primary care and when to seek emergency care because it depends on the specific situation. The participants also discussed the challenge of transitioning to a new level of care contact when the new contact may not have access to the patient’s complete history. In these sorts of cases, it was suggested that clinicians should clarify to patients that transferring to a new contact of care will involve a new assessment. In order to facilitate the care process, they also suggested that patients inform the new contact if they have presented with the same symptoms previously.

Even emergency departments and out-of-hours offices need to have a plan. It’s quite frustrating when [patients] call primary care the day after [their emergency care visit] and wonder what they should do because there’s no plan. So it’s really important that they have a plan for the immediate future. (workshop session 10, group B).

### Explain care context and purpose

The participants felt it was important that patients understood how the different parts of the healthcare system worked and what they could expect from each one. In particular, clinicians emphasised that the role of emergency care is to assess and take care of urgent care needs and that patients being treated there should not expect to receive a very detailed care plan. Explaining the role of various levels of care to patients may help them in adjusting their expectations:

…I think it’s important to convey the emergency department’s role in relation to the overall healthcare system. Or at least in relation to the concerns [patients] have right now. And it’s just about finding out what, if there’s something we urgently need to address from our perspective or if it can wait. See where it leads. And then refer them somewhere else, maybe. (workshop session 5, group B)

## Discussion

Our study shows that safety-netting is not just about one-directional communication; it involves several phases. In addition to *conveying safety-netting advice*, clinicians need to *ensure a common understanding* of the situation and *support safety-netting behaviour*. The findings detail nine safety-netting strategies that build on collaboration between clinicians, patients and carers. Although the different phases and strategies have a sequential order, iteration may be needed, suggesting that safety-netting is a cyclical rather than a linear process.

### Strengths and limitations

The longitudinal study design ensured the collection of rich data through a triangulation of data collection methods (ie, focus groups, interviews and workshops). The specificity of our participants’ backgrounds, expertise, experience and setting in relation to our aim contributed to a strengthening of the information power of our dataset.^26^ However, a limitation might be, because only one participant had carer expertise, that our findings may not fully capture the perspective of carers. Further, no parents to children were involved, and thus their specific needs which are likely to differ from other adult patients were not addressed in this study. Another consideration regarding our study participants is that the non-clinical participants were outnumbered by clinicians, which in addition to the pre-existing power imbalance may have contributed to physician voices dominating the discussions. This imbalance was partly addressed by active facilitation of workshops and focus group discussions, ensuring that all voices were heard. Further, we believe that specifically selecting highly knowledgeable and resourceful patients/carers enhanced the codesign process as they, thanks to several years of experience with regular consultations for various long-term conditions, were used to interacting with clinicians, and some of them, with researchers. However, it should also be acknowledged that given their qualities, these participants may not be representative of individuals seeking care for newly acquired symptoms and first-time encounters with clinicians, where safety-netting is particularly relevant. Thus, future studies should further elicit the preferences and experiences of individuals with less experience of clinical consultations.

The interdisciplinary author group covered relevant domain and methodological expertise. Our different backgrounds (detailed in online supplemental appendix 1) supported reflexivity during the analysis process. The intention of the codesign process was to develop safety-netting strategies that could be widely applicable in primary and emergency care settings. However, our findings do not go into depth on how the contextual differences between primary and emergency care may influence which safety-netting advice is provided and how, which is a limitation. Given that time pressure may be more pronounced in emergency care and that reconsultation is not possible, the provision of safety-netting advice may need to be more condensed. Future studies are needed to provide empirical findings about the feasibility of the proposed safety-netting strategies in both primary and emergency care, as well as possible adaptations to the two settings. Although the proposed strategies have not yet been empirically tested, several of them overlap with recommendations from previous research.^14^ Thus, we believe that our findings are transferable beyond the Swedish study context, although some of the strategies—in particular facilitating reconsultation, helping to navigate the system and explaining care context and purpose—may need to be adapted to how healthcare services are organised in the target context.

### Comparison to prior work

While prior work has provided recommendations for the content of safety-netting advice,^14 27 28^ our study focused more on the strategies for communicating advice. Our findings suggest that safety-netting involves collaboration among patients, carers and clinicians and supports the view of patients as partners in thinking through and testing diagnostic hypotheses.^29 30^ The collaborative nature of safety-netting that was highlighted in this study is consistent with a person-centred approach, integrating the dual perspectives of the patient and the clinician in the consultation.^31^ Some of the proposed safety-netting strategies overlap with previously proposed strategies promoting person-centred care. For example, *understanding patient concerns* fits in well with the ideas, concerns and expectations consultation model, which emphasises the central role of the patient’s perspective in a consultation.^31^ The ‘five cards’ guide proposed by Larsen and Neighbour^32^ further highlights the importance of summarising what the patient has said, a strategy that was also emphasised in our study. Similarly, the proposed *teach-back* method has been highlighted in previous research as effective in improving patients’ disease knowledge and self-management.^33^ The overlap of safety-netting recommendations with established consultation models has also been reported previously,^14^ indicating that safety-netting should not be viewed as an additional task in the consultation but rather as complementary components to a person-centred care approach. One complementary component is the clear documentation of safety-netting advice in the medical record, which has also been highlighted in previous recommendations.^14 27^ Thanks to online record access for patients in Sweden as well as many other countries,^34^ this practice may serve dual purposes, informing both future clinicians and the patient about the provided advice. This may be an additional consideration informing the advancement of online record access in the European Union.^35^

Some of the strategies proposed in our findings, such as *anticipating questions post consultation*, *helping to navigate the system* and *explaining the care context and purpose,* are less commonly discussed in the literature. These strategies emphasise that guidance may be needed beyond the time and scope of the clinical encounter to allow for follow-up questions and facilitate access of care at the right level of care, which is supported by the safety-netting programme theory proposed by Friedemann Smith *et al*.^14^ The need for clear and concrete advice, for example, regarding how to seek further help, was repeatedly emphasised by the study participants, a finding already well established in the existing research.^27 36^ The implementation of strategies aimed at supporting safety-netting behaviour is not merely dependent on clinicians and patients but may require organisational and policy changes (eg, to facilitate follow-up questions, reconsultation and accessible documentation). This emphasises that safety-netting is a shared responsibility between multiple stakeholders, involving clinicians, patients, carers, healthcare managers and policymakers.

### Implications for practice

We want to emphasise two main implications for practice. First, to successfully apply safety-netting strategies, clinicians need to adopt a person-centred approach that builds on collaboration. Second, safety-netting should be viewed as an iterative, rather than a linear, process. The three main safety-netting phases proposed in this study, and their related strategies, have a natural sequential order that begins with conveying safety-netting advice, followed by feedback loops to ensure common understanding, and finally the provision of support for safety-netting behaviour. The clinician may need to iterate between individual strategies and move back and forth between phases. Viewing safety-netting as ‘a dance of safety’ can remind clinicians of its cyclical and cocreated nature that requires close collaboration between an attentive leader and an engaged follower.

### Unanswered questions and future research

The usefulness of the proposed safety-netting strategies for primary and emergency care needs to be explored in clinical practice. Because these strategies are mainly targeting clinicians, future research should also focus on developing support material targeting patients and carers.

## Conclusions

Our study shows that safety-netting is more than one-directional communication of advice. It is an iterative and cocreated process between the clinician and patient, possibly supported by carers. Importantly, as the safety-netting process happens mainly after the clinical encounter, strategies for anticipating postconsultation inquiries and facilitating reconsultation are important, a care area which has not received much attention in previous studies.

## supplementary material

10.1136/bmjopen-2024-089224 online supplemental file 1

10.1136/bmjopen-2024-089224 online supplemental file 2

## Data Availability

Data are available on reasonable request.
