# Supplementary material for: Safety-netting strategies for primary and emergency care: a codesign study with patients, carers and clinicians in Sweden
Source: BMJ Open. 2024 Aug 5;14(8):e089224. doi: 10.1136/bmjopen-2024-089224 (PMC11308890; doi:10.1136/bmjopen-2024-089224)
Supplement: online supplemental file 1 [file bmjopen-14-8-s001.pdf]

Appendix 1 – Participant and research team characteristics

Table 1. Participant characteristics

ID	Role	Gender	Clinical expertise/Years of healthcare experience[1]	Participation			
				FGD	Int	WS no	Total frequency
P01	Patient	f	9 years	Yes		1	2
P02	Patient	f	missing	Yes			1
P03	Informal caregiver	m	8 years	Yes		1,2	3
P04	Patient	f	7 years	Yes			1
P05	Patient	m	10 years	Yes			1
P06	Patient	f	31 years	Yes	Yes	2,4,5	5
P07	Patient	m	35 years	Yes	Yes	2–5	6
P08	Patient	f	6 years	Yes	Yes	1,3,4	5
L01	Clinician	f	Primary care specialist	Yes	Yes	4	3
L02	Clinician	f	Primary care specialist	Yes			1
L03	Clinician	f	Primary care specialist	Yes	Yes		2
L04	Clinician	f	Emergency care specialist	Yes			1
L05	Clinician	f	Primary care specialist and emergency care resident	Yes	Yes	2–4	5
L06	Clinician	f	Primary care specialist	Yes	Yes		2
L07	Clinician	m	Primary care specialist	Yes			1
L08	Clinician	m	Primary care resident	Yes			1
L09	Clinician	f	Primary care specialist	Yes			1
L10/L11*	Clinician	m	Emergency care specialist		Yes	1	2
L12	Clinician	m	Primary care specialist			1,3,5	3
L13	Clinician	f	Primary care resident			1,2	2
L14	Clinician	f	Emergency care/paediatric care specialist			1	1
L15	Clinician	f	Primary care resident			1,3–5	4
L16	Clinician	m	Primary care resident			4	4
L17	Clinician	f	Primary care specialist			3	3
L18	Clinician	f	Primary care specialist			1,4,5	3
L19	Clinician	m	Primary care specialist			1,4,5	3

Notes. 1) For patients and informal caregivers, we report the years of experiene with routine healthcare contacts (i.e., at least one consultation per year). FGD = Focus group discussion, Int = Interview, WS no = workshop number (1-5), f = female, m = male. *One of the participants mistakenly got two ID numbers.

Research team

The research team (all female) consisted of the five co-authors and one research assistant with a nursing background who contributed to data collection (interviews and workshops) and analysis. The research team shares a devotion to quality improvement in healthcare and patient safety. CW and SR have a particular interest in digital health technologies. The authors' credentials and occupations at the time of the study were as follows: CW (PhD, associate professor in health informatics) and SR (PhD, researcher in health informatics and patient researcher) had full-time research appointments; apart from their research occupations, KPH (MD, PhD, associate professor in patient safety) worked as a pediatric emergency specialist and patient safety expert and RF (MD, PhD) as a general practitioner and quality improvement specialist; JH (MD) had just completed her medical studies and was employed as a research assistant. The research team has many years of experience with qualitative research as well as co-design studies. During data collection, the researchers briefly presented to participants their occupations as well as the main purpose of the research project. KPH and RF were professionally acquainted with some of the participating clinicians and SR with some of the participating patients/carers.
