# Supplementary material for: Safety-netting strategies for primary and emergency care: a codesign study with patients, carers and clinicians in Sweden
Source: BMJ Open. 2024 Aug 5;14(8):e089224. doi: 10.1136/bmjopen-2024-089224 (PMC11308890; doi:10.1136/bmjopen-2024-089224)
Supplement: online supplemental file 2 [file bmjopen-14-8-s002.pdf]

Appendix 2 – Overview of the co-design workshops

Table 1 provides an overview of the general time schedule and structure of the five co-design workshops. Table 2 provides an overview of the workshop aims[1], guiding questions and activities for each workshop session, and workshop outputs produced by the participants.

Table 1. General time schedule and structure of the workshops.

Time	Activities
20 min	Introduction of co-design and workshop aim<br>Participant introduction<br>Presentation of results from prior sessions
20 min	Group session 1 (2 mixed groups, including service users and clinicians)
10 min	Summary from the group session 1
5 min	Break
20 min	Group session 2 (2 mixed groups, including service users and clinicians)
10 min	Summary from the group session 2
5 min	Check-out

Table 2. Workshop aims, session details, and outputs.

		Sessions			
**WS**	**Aims**	**No**	**Guiding questions**	**Activities**	**Outputs**
1	- To create a shared idea of what safety-netting implies in the patient-clinician consultation<br>- To set goals for what safety-netting should accomplish	1<br><br>2	What does safety-netting in the patient-doctor consultation imply? Try to give concrete examples.<br><br>What would you want safety-netting to result in for the patient and the clinician? In other words, what are we trying to accomplish?	Group discussion<br><br>Group discussion	- Padlet board with participants' individual reflections collected during summary/check-out
2	- To figure out how safety-netting can be integrated practically in the patient-clinician consultation<br>- To agree on necessary content for an education aimed at supporting the use of safety-netting	3<br><br>4	How can one integrate safety-netting practically in the patient-clinician encounter?<br><br>What should an education for clinicians contain to stimulate and support the use of safety-netting? Theory & practice	Group discussion<br><br>Group discussion	- Participants' individual summaries of key points (shared in the chat during summary/check-out)
3	- To collaboratively design a format for safety-netting advice based on a number of symptom-based cases	5	Based on a case description, design a proposal for safety-netting advice	Design exercise (group A: primary care case; group B: emergency care case)	- Word documents with suggestions for safety-netting

[1] Note that the co-design workshops had a dual purpose to develop both safety-netting strategies and educational content for a safety-netting course for clinicians. The full details are provided here for the purpose of comprehensiveness. However, in the analysis for this study, we have focused on safety-netting strategies and have left out data related specifically to educational content.

WS	Aims	Sessions<br>No	 Guiding questions	 Activities	Outputs
		6	Look at the other group's suggestion and discuss/complement	Design exercise (group A: primary care case; group B: emergency care case)	advice (created during the design exercise)<br>- Participants' individual take-home messages (shared in the chat during summary/check-out)
4	- To collaboratively design a proposal for a safety-netting education<br>- To make suggestions for how to deliver content	7<br><br>8	Draft an educational curriculum (a theory group and a practice group)<br><br>Further develop the educational curriculum	Design exercise (group A: theory; group B: practice)<br><br>Design exercise (group A: theory; group B: practice)	- Mindmaps with suggestions for a safety-netting curriculum (created during group exercise)<br>- Participants' individual summaries of key points (shared in the chat during summary/check-out)
5	- To further develop the education for clinicians<br>- To together agree on main learning outcomes for a safety-netting education	9<br><br>10	How should a clinician who has taken the course behave?<br><br>What kind of situation would you want the role plays to highlight?	Group discussion<br><br>Group discussion (group A: good example, group B: bad example)	- Participants' individual suggestions of a Swedish term for safety-netting and a name for the course (shared in the chat during summary/check-out)
