# Supplementary material for: Safety-netting strategies for primary and emergency care: a codesign study with patients, carers and clinicians in Sweden
Source: BMJ Open. 2024 Aug 5;14(8):e089224. doi: 10.1136/bmjopen-2024-089224 (PMC11308890; doi:10.1136/bmjopen-2024-089224)
Supplement: Supplementary file 3 [file bmjopen-2024-089224.reviewer_comments.pdf]

PEER REVIEW HISTORY

BMJ Open publishes all reviews undertaken for accepted manuscripts. Reviewers are asked to complete a checklist review form (**http://bmjopen.bmj.com/site/about/resources/checklist.pdf**) and are provided with free text boxes to elaborate on their assessment. These free text comments are reproduced below.

ARTICLE DETAILS

**TITLE (PROVISIONAL)**	Safety-netting strategies for primary and emergency care: A co-design study with patients, carers, and clinicians in Sweden
**AUTHORS**	Wannheden, Carolina; Hagman, Johanna; Riggare, Sara; Pukk Härenstam, Karin; Fernholm, Rita

VERSION 1 – REVIEW

**REVIEWER**	Friedemann Smith, Claire<br>University of Oxford, Nuffield Department of Primary Care Health Sciences
**REVIEW RETURNED**	18-Jun-2024

**GENERAL COMMENTS**	Thank you for asking me to review this well written and interesting paper. As the authors comment, the patient perspective is not often captured in studies on safety-netting and so this paper provides a valuable addition to the literature. I have a few comments as listed below and if these were addressed I would recommend that the paper is published.<br><br>Page 2 line 53-54 – sentence needs clarification.<br>Page 3 this paper https://qualitysafety.bmj.com/content/31/7/541.abstract is relevant to the points made in lines 19-24.<br>Page 3 this paper https://pubmed.ncbi.nlm.nih.gov/35858826/ is relevant to the point made in line24-25.<br>Methods – consent process is described but please could the details of any ethics application be given.<br>Discussion – is it possible to mention or discuss how the different setting in which safety-netting advice might affect the conclusions. For example, allowing for feedback and follow-up questions might be possible in general practice but was it also mentioned/how would it be managed in emergency or out of hours care?<br>The authors also briefly mention continuity of care (or lack thereof) was this only mentioned in the context of the patient being able to see the same clinician if they re-consult or was there any mention of it in terms of how other clinicians are informed of safety-netting advice given previously?

**REVIEWER**	Oostenbrink, Rianne<br>Erasmus MC - Sophia Children's Hospital
**REVIEW RETURNED**	20-Jun-2024

**GENERAL COMMENTS**	The authors presents important work on safetynetting<br>study design and methods are appropriate<br>results well described and discussed

	My key comments is that it is a mixed approach for primary and ED care, which may affect the way results are presented, and the discussionsection (limitations, and implications).<br>although I see some overlap (new complaints without preceeding history), also in primary care patients may have chronic conditions where to consult the clinician for, which contrast for ED care. ED care is on excluding emergency care requiring conditions, and missed diagnoses may have different consequences than the conditions consulted for in primary care (where the missed diagnosis may have a less fast deteriorating course?). next, the setting is different in time-pressure perspective.<br>although I also see some overlap, I think the authors should better discuss potential need for different approaches in the two settings, in terms of type of information, but also on length of the consultation spent to the safetynet procedure. How can we make SN strategies feasible in ED setting.<br>next, patients in ED are more 'unknown' to the consulting clinician than in primary care. or do the authors mean ED and primary emergency settings (shift consultations), then they should make this more clear. In this sense it also would be interesting to understand the background of both clinicians and patients on type on diseases (chronic versus acute) etc . How could this have influenced the results, and what gaps remain?<br><br>A second point is to better reflect on existing literature on safetynetting. Are the themes different from previous literature, and what do they contribute? New items, more details? The results are more now reflected on communicationstrategies in consultations in general, but what about specific SN strategies.<br>I appreciate the educational strategies of re-asking, but it’s feasibility should be discussed in the time-pressure setting of the ED<br><br>minor comments:<br>check language, e.g. for my understanding UK word 'film' (p4)isnot referrring to 'what the authors intend ('movie'?), so perhaps a broader check needs to be performed (although I am not a native speaker as well).<br>I appreciate the educational strategies of re-asking, but it’s feasibility should be discussed in the time-pressure setting of the ED<br>The authors report that expansion can be in the region around the hospital of research (p12 bottom). But what about generalisation to Europe? To what settings does and does it not fit?<br>table 2: could for patient experiences be added if they suffer from a condition and type/group (e.g. acute vs chronic)

**REVIEWER**	Neill, Sarah<br>University of Plymouth, School of Nursing and Midwifery
**REVIEW RETURNED**	27-Jun-2024

**GENERAL COMMENTS**	Lovely to see a report of a co-design project focussing on safety-netting. It reads well. You have referred to a preceding paper in the methods section but it would be helpful to add details of the ethical approval secured if this paper is to stand alone.<br>You highlighted the power imbalance which exists during consultations yet have not extended this thinking to the conduct of the project. Mixing patients and clinicians, especially when patients are outnumbered by clinicians, will inevitably lead to clinician voices dominating. This limitation need to be more clearly

	stated in the limitations section. There also seemed to be no parents with children involved - if this is the case then this limitation should also be acknowledged as parents safety-netting needs are likely to be different to adult patients.<br>Please provide a brief overview of the structure of Swedish health services in the background to the paper. This will help readers to contextualise the findings.<br>Please add the characteristics of the participant to all the quotations included.

VERSION 1 – AUTHOR RESPONSE

Reviewer: 1
Dr. Claire Friedemann Smith, University of Oxford
Comments to the Author:
Thank you for asking me to review this well written and interesting paper. As the authors comment, the patient perspective is not often captured in studies on safety-netting and so this paper provides a valuable addition to the literature. I have a few comments as listed below and if these were addressed I would recommend that the paper is published.

Response: Thank you very much for your review and helpful comments!

Page 2 line 53-54 – sentence needs clarification.

Response: Thank you, we have now clarified this sentence and hope that it reads better (p. 2, 1st paragraph).

Page 3 this paper https://qualitysafety.bmj.com/content/31/7/541.abstract is relevant to the points made in lines 19-24.

Response: Thank you very much for highlighting this! The realist review is indeed very relevant to our study and we have now added this reference in the introduction (p. 3, ref 15) and refer to it at multiple locations in the discussion.

Page 3 this paper https://pubmed.ncbi.nlm.nih.gov/35858826/ is relevant to the point made in line24-25.

Response: Thank you, we have added a reference to the suggested paper (p.3, ref 16).

Methods – consent process is described but please could the details of any ethics application be given.

Response: A statement has been added at the end of the manuscript (under the heading “Ethical Approval”) and we have also moved the details regarding the consent process there (p. 16).

Discussion – is it possible to mention or discuss how the different setting in which safety-netting advice might affect the conclusions. For example, allowing for feedback and follow-up questions might be possible in general practice but was it also mentioned/how would it be managed in emergency or out of hours care?

Response: Thank you for raising this. We realize that our findings do not go into depth on how the contextual differences between primary and emergency care influence the application of safety-

netting strategies. We have now added some reflections about this in the second paragraph of the Strengths and limitations section (p. 12)

The authors also briefly mention continuity of care (or lack thereof) was this only mentioned in the context of the patient being able to see the same clinician if they re-consult or was there any mention of it in terms of how other clinicians are informed of safety-netting advice given previously?

Response: The discussion of continuity that you are referring to (p. 10, Facilitate re-consultation) concerned relational continuity, which we have now clarified. However, information continuity was also addressed as participants advocated clearly documented safety-netting advice that could be helpful for both patients and clinicians (p. 9, Anticipate questions post-consultation).

Reviewer: 2
Mrs. Rianne Oostenbrink, Erasmus MC - Sophia Children's Hospital
Comments to the Author:
The authors presents important work on safetynetting
study design and methods are appropriate
results well described and discussed

Response: Thank you very much for your review and helpful comments!

My key comments is that it is a mixed approach for primary and ED care, which may affect the way results are presented, and the discussionsection (limitations, and implications).
although I see some overlap (new complaints without preceeding history), also in primary care patients may have chronic conditions where to consult the clinician for, which contrast for ED care. ED care is on excluding emergency care requiring conditions, and missed diagnoses may have different consequences than the conditions consulted for in primary care (where the missed diagnosis may have a less fast deteriorating course?). next, the setting is different in time-pressure perspective. although I also see some overlap, I think the authors should better discuss potential need for different approaches in the two settings, in terms of type of information, but also on length of the consultation spent to the safetynet procedure. How can we make SN strategies feasible in ED setting.
next, patients in ED are more 'unknown' to the consulting clinician than in primary care. or do the authors mean ED and primary emergency settings (shift consultations), then they should make this more clear. In this sense it also would be interesting to understand the background of both clinicians and patients on type on diseases (chronic versus acute) etc . How could this have influenced the results, and what gaps remain?

Response: Thank you for these important comments. We acknowledge that our study does not go into depth on how the contextual differences between primary and emergency care influence the application of safety-netting strategies. We have now added some reflections about this in the second paragraph of the Strengths and limitations section (p. 12). Further, in the first paragraph (same section), we have expanded our reflections about the non-clinical participants' background and how this may have affected the results. Participants were not specifically recruited for their type of health conditions (e.g. acute versus chronic), but rather for their experience with regular health consultations, which suggests that the participants had experience living with long-term conditions. We believe that this may have benefited the co-design process but may also be a limitation as they were not representative of patients presenting with new symptoms and in consultations with clinicians that they may not have met previously (i.e., patients unknown to the consulting clinician). This limitation is now added.

A second point is to better reflect on existing literature on safetynetting. Are the themes different from

previous literature, and what do they contribute? New items, more details? The results are more now reflected on communicationstrategies in consultations in general, but what about specific SN strategies.
I appreciate the educational strategies of re-asking, but it's feasibility should be discussed in the time-pressure setting of the ED

Response: Thank you for pointing this out. In the Discussion section titled Comparison to prior work (p. 13), we have now clarified that our findings are not primarily focused on proposing new recommendations about safety-netting content (which has been done in prior work), but rather focusing on the strategies for communicating safety-netting advice. Nevertheless, we acknowledge that our findings overlap with existing literature on safety-netting recommendations and we have now added more comparisons to prior work focusing specifically on safety-netting.

minor comments:
check language, e.g. for my understanding UK word 'film' (p4)isnot referrring to 'what the authors intend ('movie'?), so perhaps a broader check needs to be performed (although I am not a native speaker as well).

Response: Thank you for your comment. The manuscript has been professionally edited. The term "film" is based on the experience-based co-design method, which uses the term "trigger film". We have now adjusted the text to use the term "trigger film" for increased clarity (p. 4).

I appreciate the educational strategies of re-asking, but it's feasibility should be discussed in the time-pressure setting of the ED

Response: Thank you, as our findings unfortunately do not yet provide answers regarding feasibility, this has now been addressed as a limitation and area for future research in the Strengths and limitations section (p. 12).

The authors report that expansion can be in the region around the hospital of research (p12 bottom). But what about generalisation to Europe? To what settings does and does it not fit?

Response: This has also been addressed in the last two sentences of the Strengths and limitations section (p. 12). Given the overlap of our findings with previous research outside of Sweden, we believe that our findings are transferable beyond the Swedish study context, although some of the strategies – in particular facilitating re-consultation, helping to navigate the system, and explaining care context and purpose – may need to be adapted to how healthcare services are organized in the target context.

table 2: could for patient experiences be added if they suffer from a condition and type/group (e.g. acute vs chronic)

Response: We assume that you are referring to the patient characteristics table in Appendix 1. In the notes to the table, we have clarified the following: "For patients and informal caregivers, we report the years of experience with routine healthcare contacts (i.e., at least one consultation per year)." This suggests that patients had long-term conditions, without specifying if they would be considered chronic. We hope that this clarification is acceptable.

Reviewer: 3
Prof. Sarah Neill, University of Plymouth
Comments to the Author:

Lovely to see a report of a co-design project focussing on safety-netting. It reads well. You have referred to a preceding paper in the methods section but it would be helpful to add details of the ethical approval secured if this paper is to stand alone.

Response: Thank you for your review and helpful comments! A statement has been added at the end of the manuscript (p. 16, under the heading “Ethical Approval”) and we have also moved the details regarding the consent process there.

You highlighted the power imbalance which exists during consultations yet have not extended this thinking to the conduct of the project. Mixing patients and clinicians, especially when patients are outnumbered by clinicians, will inevitably lead to clinician voices dominating. This limitation need to be more clearly stated in the limitations section.

Response: Thank you for raising this. We have added reflections on the power imbalance and how we addressed this in the first paragraph of the Strengths and limitations section (p. 12).

There also seemed to be no parents with children involved - if this is the case then this limitation should also be acknowledged as parents safety-netting needs are likely to be different to adult patients.

Response: Thank you, this is correct. We have now also added this as a limitation (p. 12).

Please provide a brief overview of the structure of Swedish health services in the background to the paper. This will help readers to contextualise the findings.

Response: Thank you for suggesting this. We have now added a section titled Swedish healthcare context under the Methods section (p. 3-4). We hope that this is helpful.

Please add the characteristics of the participant to all the quotations included.

Response: Unfortunately, we cannot add individual participant characteristics to the quotations as these are extracted from transcribed workshops where individual participant voices were not distinguished (as is common in the analysis of focus group discussions). From the context, we could nevertheless determine whether the quotation was from a clinician or a patient/caregiver, wherefore we have provided this information.

VERSION 2 – REVIEW

**REVIEWER**	Oostenbrink, Rianne<br>Erasmus MC - Sophia Children's Hospital
**REVIEW RETURNED**	19-Jul-2024

**GENERAL COMMENTS**	Thanks for revising the manuscript. The message of the paper is much better positioned. I think this is a worthfully contribution to the field of safety netting
